# Evaluation of Analytical Agreement Between High-Performance Liquid Chromatography and Capillary Electrophoresis for Haemoglobin A_2_ Quantification: A Meta-Analysis

**DOI:** 10.21315/mjms-11-2025-818

**Published:** 2026-04-30

**Authors:** Yongzhong Wei, Mingze Gu, Yuxin Su, Jiayun Xi, Yuqing Ren, Zhongshuang Hu

**Affiliations:** Institute of Biomaterials and Biomedicine, School of Food and Pharmacy, Shanghai Zhongqiao Vocational and Technical University, Shanghai, People’s Republic of China

**Keywords:** haemoglobin A_2_, HPLC, capillary electrophoresis, bias, hemoglobinopathy

## Abstract

**Background:**

Haemoglobin A_2_ (HbA_2_) measurement is essential for diagnosing β-thalassemia carriers, yet even small analytical biases may affect clinical interpretation near the diagnostic threshold of 3.5%. High-performance liquid chromatography (HPLC) and capillary electrophoresis (CE) are widely used, but their agreement remains uncertain. The study aimed to systematically evaluate the measurement bias between HPLC and CE in HbA_2_ quantification.

**Methods:**

A systematic review and meta-analysis were conducted in accordance with the Preferred Reporting Items for Systematic Reviews and Meta-Analyses (PRISMA) 2020 guidelines. Eligible studies directly compared HbA_2_ values measured by HPLC and CE in the same samples. Data on mean bias, standard deviation (SD), and limits of agreement (LoA) were extracted. A random-effects model was used to pool mean bias, and subgroup analyses were performed for normal individuals, β-thalassemia carriers, and haemoglobin variant carriers. Sensitivity analysis was conducted to assess robustness.

**Results:**

Ten studies with 1,881 paired measurements were included. After excluding datasets with clear co-elution interference, mainly haemoglobin E (HbE) and haemoglobin Hope (Hb Hope), the pooled mean bias was 0.16% (95% confidence interval [CI]: 0.03, 0.29), with high heterogeneity (*I*^2^ = 99.5). Subgroup analysis showed a mean bias of 0.37% (95% CI: 0.24, 0.50) in healthy individuals and 0.39% (95% CI: 0.20, 0.59) in β-thalassemia carriers. Sensitivity analysis confirmed the stability of the results.

**Conclusion:**

HPLC and CE demonstrate systematic but clinically relevant differences in HbA_2_ measurement, particularly near diagnostic thresholds. HbA_2_ measurement results obtained from HPLC and CE should not be used interchangeably, and method-specific interpretation and harmonisation efforts are warranted.

## Introduction

Haemoglobin A_2_ (HbA_2_) is a minor adult haemoglobin fraction, normally constituting about 2% to 3% of total haemoglobin. Despite its small proportion compared to the predominant haemoglobin A (HbA), HbA_2_ plays a pivotal role in the diagnosis of haemoglobinopathies (1). Elevated HbA_2_ levels are a well-established biomarker for identifying β-thalassemia carriers and are routinely used in population-based screening programmes worldwide (1–3). While β-thalassemia remains the primary clinical condition associated with abnormal HbA_2_ expression, recent studies suggest that HbA_2_ quantification may also aid in diagnosing other disorders, such as sickle cell disease and complex haemoglobin variants, in which abnormal HbA_2_ levels often reflect underlying β-chain defects or fusion events (4, 5). These broader applications highlight the need for robust standardised HbA_2_ measurements across clinical settings.

Accurate quantification of HbA_2_ is crucial, as even small differences can impact diagnostic classification. The absolute difference in HbA_2_ levels between β-thalassemia carriers and non-carriers is typically modest (about 1% to 3%), yet this margin straddles the commonly used diagnostic threshold of 3.5% (6, 7). Consequently, even minor analytical biases may shift results across this threshold, potentially leading to misclassification, inappropriate patient counselling, or failure to detect carriers. This is especially concerning near the diagnostic threshold, where both high precision and accuracy are critical (8, 9).

Among the available analytical techniques for HbA_2_ quantification, high-performance liquid chromatography (HPLC) and capillary electrophoresis (CE) are the most widely implemented in clinical laboratories (8). Ion-exchange HPLC separates haemoglobin fractions according to differences in ionic charge, using cation-exchange stationary phases to achieve high resolution and reproducibility (8). This approach is well-suited for high-throughput workflows and has long been the mainstay of HbA_2_ measurement. In contrast, CE separates haemoglobin variants according to their electrophoretic mobility in a capillary under a high-voltage electric field (8). Differences in charge-to-size ratio and tertiary structure enable efficient resolution of haemoglobin components. Compared with ion-exchange HPLC, CE typically provides comparable or slightly shorter analysis time, while also improving the resolution of certain variants that may overlap with HbA_2_ on HPLC (10).

Although both HPLC and CE are widely used for HbA_2_ quantification, concerns remain regarding the consistency of results across platforms. Moreover, the presence of haemoglobin variants or abnormal fractions can challenge the separation or peak identification in both techniques, potentially affecting reported HbA_2_ values. These factors raise important questions about whether results obtained by the two methods can be interpreted interchangeably in clinical or screening contexts.

To address this issue, this study conducted a meta-analysis focusing on measurement bias between HPLC and CE in the quantification of HbA_2_, using pooled mean differences and limits of agreement (LoA) to assess intermethod consistency. The findings indicate that the two methods yield systematic differences that are clinically relevant near the diagnostic threshold, underscoring the need for method-specific interpretation and improved standardisation efforts.

## Methods

### Study Design and Registration

This meta-analysis aimed to evaluate the measurement bias between HPLC and CE for the quantification of HbA_2_. The study was conducted in accordance with the Preferred Reporting Items for Systematic Reviews and Meta-Analyses (PRISMA) 2020 guidelines (11). The review protocol was prospectively registered in the International Prospective Register of Systematic Reviews (PROSPERO) under the registration number CRD420251104147.

### Search Strategy and Study Selection

A comprehensive literature search was conducted in PubMed, Embase, Scopus, and Web of Science from inception of each database through July 2025. The search strategy combined terms related to HbA_2_ (e.g., “HbA_2_” and “haemoglobin A_2_”), analytical methods (e.g., “high-performance liquid chromatography”, “capillary electrophoresis”, “HPLC”, and “CZE”), and performance evaluation (e.g., “comparison”, “agreement”, “bias”, and “validation”). Only studies published in English were considered.

Studies were considered eligible if they were original research articles that directly compared HbA_2_ levels measured by HPLC and CE in the same set of human samples and reported quantitative data sufficient to assess measurement bias, agreement, or correlation between the two methods. Both healthy individuals, defined as those without known blood disorders or diabetes, and individuals with hemoglobinopathies such as β-thalassemia, were included. Eligible study designs encompassed randomised and non-randomised investigations conducted in clinical or academic laboratory settings. In contrast, studies were excluded if they were reviews, conference abstracts without extractable data, animal studies, or if they used methods other than HPLC or CE for HbA_2_ quantification.

The authors (YW and MG) independently screened titles, abstracts, and full texts. Disagreements were resolved by discussion or, when necessary, by consultation with ZH.

### Quality Assessment

The methodological quality of the included studies was assessed using the Quality Assessment of Diagnostic Accuracy Studies 2 (QUADAS-2) tool, which evaluates four domains: patient selection, index test, reference standard, and flow and timing (12).

### Data Extraction

For each included study, the following information was extracted: first author, publication year, study country, sample size, patient population (e.g., healthy individuals, β-thalassemia carriers, or haemoglobin variant cases), and the specific instruments used for HPLC and CE measurements. The primary outcomes of interest were the mean difference (bias) between HPLC and CE measurements of HbA_2_, the standard deviation (SD) of the bias, and the 95% LoA (13, 14).

To ensure consistency across studies, all biases were expressed as HPLC minus CE values. If a study reported the bias in the reverse direction, the sign was inverted accordingly. In studies that reported only raw measurement data for HPLC and CE, the mean bias and SD were manually calculated. When the SD of paired differences was not reported, it was derived from the published LoA according to the Bland-Altman method. Assuming an approximately normal distribution of paired differences, the SD was calculated as: SD = (Upper LoA − Lower LoA) / (2 × 1.96). When 95% confidence interval (CI) for the mean bias was reported instead of SD, SD was back-calculated assuming a normal distribution and known sample size using the formula for CI.

To ensure consistency, when a single study reported multiple subgroups that could reasonably be combined into a single dataset (e.g., stratification by variant type or demographic characteristics), the results were pooled prior to meta-analysis. Mean bias values were combined using sample size weighting, while SDs were reconstructed from reported LoA and then pooled using a variance weighted approach that accounts for both within and between subgroup variability. This approach allowed to include all relevant information without overweighting studies that reported multiple related estimates.

Data extraction was independently performed by two authors (YW and MG) using a standardised template. Any discrepancies were resolved through discussion.

### Statistical Analysis

All statistical analyses were performed using Stata version 18.0 (StataCorp, Texas, US). A random-effects model (DerSimonian and Laird method) was used to pool mean differences between HPLC and CE measurements. Heterogeneity was assessed using the *I*^2^ statistic, with *I*^2^ > 50% indicating substantial heterogeneity. Subgroup analysis was performed based on patient characteristics and assay platform. Sensitivity analysis was performed by sequentially omitting each included study (leave-one-out method) to examine the robustness of the pooled estimates. Potential publication bias was not formally assessed using funnel plots or Egger’s test because the number of eligible studies was limited, which reduced statistical power and the interpretability of these approaches.

## Results

### Study Selection and Characteristics

A total of 494 records were retrieved from the initial database search. After removing duplicates and screening titles, abstracts, and full texts, 10 studies met the inclusion criteria and were included in the final meta-analysis (Figure 1). The included studies were published between 2009 and 2024, encompassing 1,881 paired HbA_2_ measurements comparing HPLC and CE-based methods. A wide range of population characteristics were evaluated, including normal, β-thalassemia, variants, such as haemoglobin S (HbS) and haemoglobin D (HbD), representing the complex pathogenic situations (Table 1). Moreover, multiple HPLC devices were involved, mainly including Bio- Rad Variant II and Trinity Premier. All CE-based devices included in the analysis were manufactured by Sebia (Table 1).

### Meta-analysis

Co-elution with common variants, such as HbE on the HPLC platform, is a well-recognised cause of artefactual HbA_2_ overestimation. Therefore, datasets with clear evidence of such overlap were excluded from the pooled analysis. After excluding datasets with clear evidence of co-elution (predominantly HbE and Hb Hope), the pooled mean bias between HPLC and CE was 0.16% (95% CI: 0.03, 0.29), with substantial heterogeneity (*I*^2^ = 99.5) (Figure 2). This indicates that even in the absence of major variant interference, systematic differences between the two methods remain. The analysis was conducted after exclusion of datasets with evident HbE or Hb Hope coelution. Each horizontal line shows the 95% CI for a single dataset, with the square size proportional to the study weight. The pooled effect size is represented by a diamond, with its width corresponding to the 95% CI. The term “excluded” indicates that certain datasets were automatically excluded during model fitting because of zero or invalid SE estimates. Mean bias was defined as HPLC – CE; positive values indicate higher HbA_2_ measured by HPLC, whereas negative values indicate higher HbA_2_ measured by CE.

For the normal population subgroup, the pooled mean bias was 0.37% (95% CI: 0.24, 0.50), with high heterogeneity across studies (*I*^2^ = 93.4). Despite the relatively consistent direction of effect, the variability among studies suggests that additional factors may influence method agreement in otherwise healthy populations.

Among β-thalassemia carriers, the pooled mean bias was 0.39% (95% CI: 0.20, 0.59), again accompanied by considerable heterogeneity (*I*^2^ = 88.2). Given that diagnostic thresholds typically fall around 3.5% HbA_2_, these findings highlight the potential clinical impact of method-dependent variation in this subgroup. For the β-chain variant subgroup (including HbS, HbD, and Hb Lepore), the pooled mean bias was close to zero (0.02%; 95% CI: 0.01, 0.04) with relatively narrow CIs.

In the analysis, a small number of datasets were excluded by the software because variance estimation yielded non-positive standard errors. This reflects the numerical instability of heterogeneity estimation in small subgroups rather than issues with the underlying data.

In addition, this study performed exploratory subgroup analysis stratified by HPLC platform manufacturer. Both the Premier and Bio-Rad Variant systems showed a consistent positive bias relative to CE, with pooled mean differences of 0.23% (95% CI: 0.02, 0.44) and 0.10% (95% CI: 0.08, 0.12), respectively. These findings suggest that inter-platform discrepancies are not limited to specific devices.

### Sensitivity Analysis and Publication Bias

To assess the robustness of the pooled estimate, a leave-one-out sensitivity analysis was performed. Sequential exclusion of each study produced pooled bias estimates ranging from 0.06% to 0.15%. The corresponding 95% CI all overlapped with that of the overall pooled estimate, and none of the exclusions materially altered the direction or magnitude of the effect. For example, excluding study 1 yielded a pooled bias of 0.1112% (95% CI: −0.0100, 0.2224), while excluding study 6 yielded 0.06% (95% CI: −0.0700, 0.1900). This indicates that the observed inter-platform differences are not driven by any single study and that the results are stable and robust.

## Discussion

Accurate quantification of HbA_2_ is critical for the diagnosis and screening of β-thalassemia carriers (2). Both HPLC and CE-based assay devices are widely utilised in clinical laboratories, often applying the same diagnostic cut-off for β-thalassemia trait (commonly 3.5%), and a limited number of studies have compared these two methods. The present study systematically evaluated the measurement bias between HPLC and CE for HbA_2_ quantification by performing a meta-analysis.

To minimise the confounding effect of obvious co-elution (e.g., HbE and Hb Hope), datasets with such interference were excluded from the pooled analysis, which still revealed a statistically significant residual systematic bias of 0.16% (95% CI: 0.03, 0.29). Among the normal population, the pooled mean bias was 0.37% (95% CI: 0.24, 0.50), while for β-thalassemia carriers it was 0.39% (95% CI: 0.20, 0.59), and for β-chain variants it was 0.02% (95% CI: 0.01, 0.04). Although these values are modest in absolute terms, they are clinically significant, particularly as they approach the critical 3.5% diagnostic threshold (24, 25). Moreover, the very high heterogeneity observed in both subgroups underscores that the variability between HPLC and CE is systematic, making it crucial to consider this in clinical interpretation. In summary, the findings in this study demonstrate that the two platforms yield systematically different results and should not be considered interchangeable.

The HPLC and CE devices rely on different separation principles, which may represent a key source of disagreement between the two methods (9, 10). The HPLC separates haemoglobin fractions based on ionic interactions with the stationary phase (ion-exchange chromatography) (26). While ion-exchange HPLC generally provides high resolution for HbA_2_ fractionation, it can be susceptible to co-elution with certain haemoglobin variants (e.g., HbE, HbD-Punjab, and Hb Lepore) when their retention times overlap with that of HbA_2_, potentially causing overestimation of HbA_2_ in affected samples (5, 9, 27, 28). Moreover, the extent of overlap for a given variant can vary across HPLC platforms. For instance, HbE has repeatedly been shown to co-elute with HbA_2_ on the Bio-Rad Variant system, leading to substantial overestimation (29). By contrast, on the Trinity system, HbE shows only partial overlap, and the reported HbA_2_ result is considered more reliable (30). Even within the same analytical principle, significant inter-platform bias may therefore persist. These observations further underscore the importance of cross-platform harmonisation efforts and the potential role of reference methods in minimising diagnostic discrepancies.

In contrast, CE distinguishes haemoglobin species according to their electrophoretic mobility, which often provides better resolution of certain variants that co-elute on ion-exchange HPLC. Although datasets with clear Hb Hope interference were excluded from pooled analysis, CE measurements may still be susceptible to subtle baseline or migration-zone effects in the presence of rare variants (30, 31). Thus, differences in the underlying separation physics explain much of the method-dependent disagreement in HbA_2_ quantification, underscoring the need for method-specific validation and awareness of variant-related interferences when interpreting borderline HbA_2_ results.

To minimise the confounding effect of pronounced co-elution, particularly from HbE and Hb Hope, the present study excluded datasets with clear evidence of significant overlap. However, a small but statistically significant and clinically relevant systematic pooled bias was still observed, including in the normal population (Figure 3). This suggests that inter-platform differences cannot be fully explained by overt co-elution and that additional factors contribute to the observed discrepancies. Beyond variant-related interference, methodological differences also arise from how HPLC and CE operationally define the HbA_2_ fraction. The CE assigns HbA_2_ to a migration-zone, whereas ion-exchange HPLC uses retention-time windows, and the two approaches may not classify minor fractions identically (8, 32). Furthermore, differences in how manufacturers process chromatograms or electropherograms (e.g., baseline handling and peak integration rules) may also contribute, although detailed evidence is scarce in the published literature. These discrepancies emphasise that HbA_2_ results from different platforms are not strictly interchangeable and further support the International Federation of Clinical Chemistry and Laboratory Medicine (IFCC) initiative to establish a mass spectrometry-based reference procedure as a chemically defined anchor for harmonisation (8).

Figure 3 shows the pooled mean bias in three subgroups: i) normal population; ii) β-thalassemia carriers; and iii) individuals with haemoglobin variants. Each horizontal line represents the 95% CI for a single dataset, with the square size proportional to the study weight. The pooled effect size is depicted by a diamond, whose width reflects the 95% CI. “Excluded” means certain datasets were automatically excluded during model fitting because of zero or invalid SE estimates. Mean bias was defined as HPLC – CE; positive values indicate higher HbA_2_ measured by HPLC, whereas negative values indicate higher HbA_2_ measured by CE.

Calibration and standardisation practices represent another major source of inter-method variability in HbA_2_ measurement. Although the World Health Organization (WHO) has established an international reference reagent for HbA_2_, provided by the National Institute for Biological Standards and Control (NIBSC 89/666), most commercially available assays are not directly traceable to this material. This reflects both the methodological challenges of aligning operationally defined fractions with a chemically defined reference and the limited regulatory pressure compared with more standardised analytes such as HbA1c. Consequently, instrument- and manufacturer-specific biases have persisted across platforms (8, 9). Recent harmonisation efforts, including the development of IFCC reference measurement procedures, certified reference reagents, and consensus guidance documents, have helped reduce some of the between-method differences, yet uptake and implementation remain uneven across laboratories and manufacturers (33, 34).

For HbA_2_ in particular, national and international external quality assessment (EQA) surveys and pilot studies have repeatedly demonstrated inter-laboratory and inter-method biases, which motivated the International Council for Standardization in Haematology (ICSH) to issue specific recommendations for performance assessment and to encourage broader adoption of reference materials and method verification (8, 30, 35). In practice, manufacturer-level calibration algorithms and lot-to-lot reagent variability can interact with the underlying analytic principle (ion-exchange, CE, or boronate-based HPLC) to amplify or attenuate observed biases in certain settings, a complication that persists despite the availability of reference reagents. Thus, although international standardisation initiatives and the availability of reference materials were intended to improve cross-platform comparability, calibration-related differences remain an important contributor to the residual heterogeneity observed in HbA_2_ meta-analysis (35–38).

From a clinical perspective, the limited interchangeability demonstrated by the present study’s findings between HPLC and CE for HbA_2_ quantification has important implications for thalassaemia screening programmes, especially in high-prevalence regions (2). Even small systematic biases around the commonly used diagnostic threshold (~3.5% for β-thalassaemia) may shift results across the cut-off, leading to false positives or false negatives and affecting counselling and reproductive risk assessment (2, 35). Laboratories should therefore avoid naive cross-platform pooling of results and instead apply method-specific reference limits, local verification or cross-calibration, and cautious interpretive language when reporting borderline values (8). In settings with a high-prevalence of structural variants or modifying genotypes, reliance on a single platform increases the risk of misclassification; for borderline or discordant cases, orthogonal confirmation, such as repeat testing using a different separation principle, targeted molecular assays, or referral to a specialist laboratory, is recommended to reduce diagnostic uncertainty and enable appropriate genetic counselling (1, 5, 39).

In addition to bias, the interpretation of chromatograms or electropherograms poses further clinical challenges, particularly when unexpected variant peaks or atypical patterns are present. Accurate recognition and classification of such peaks require significant expertise, and misinterpretation can compound the risk of diagnostic errors. Recent studies have suggested that computational algorithms and artificial intelligence, based on pattern recognition, may aid in automating variant detection and improving the consistency of chromatogram interpretation (40).

This study has several strengths, including a comprehensive literature search, strict inclusion criteria, and subgroup exploration based on both clinical and analytical variables. However, limitations must be acknowledged. The number of included studies was modest, with limited representation of some HPLC principles or specific variants. Many studies lacked complete quantitative agreement metrics, such as Bland-Altman plots, restricting more nuanced statistical evaluation. Publication bias assessment was constrained by the small number of studies in most subgroups. In addition, publication bias was suggested in the variant subgroup, further limiting certainty in this group.

## Conclusion

This meta-analysis demonstrated a systematic analytical bias between HPLC and CE in haemoglobin A_2_ (HbA_2_) quantification. After excluding datasets with clear co-elution interference, the two methods still showed clinically relevant differences, particularly in normal individuals and β-thalassaemia carriers near the diagnostic threshold. Therefore, HbA_2_ results obtained using HPLC and CE should not be used interchangeably. Laboratories should apply method-specific interpretation, perform local verification where possible, and consider confirmatory testing for borderline or discordant cases, especially in the presence of haemoglobin variants.

## Figures and Tables

**Figure 1:**
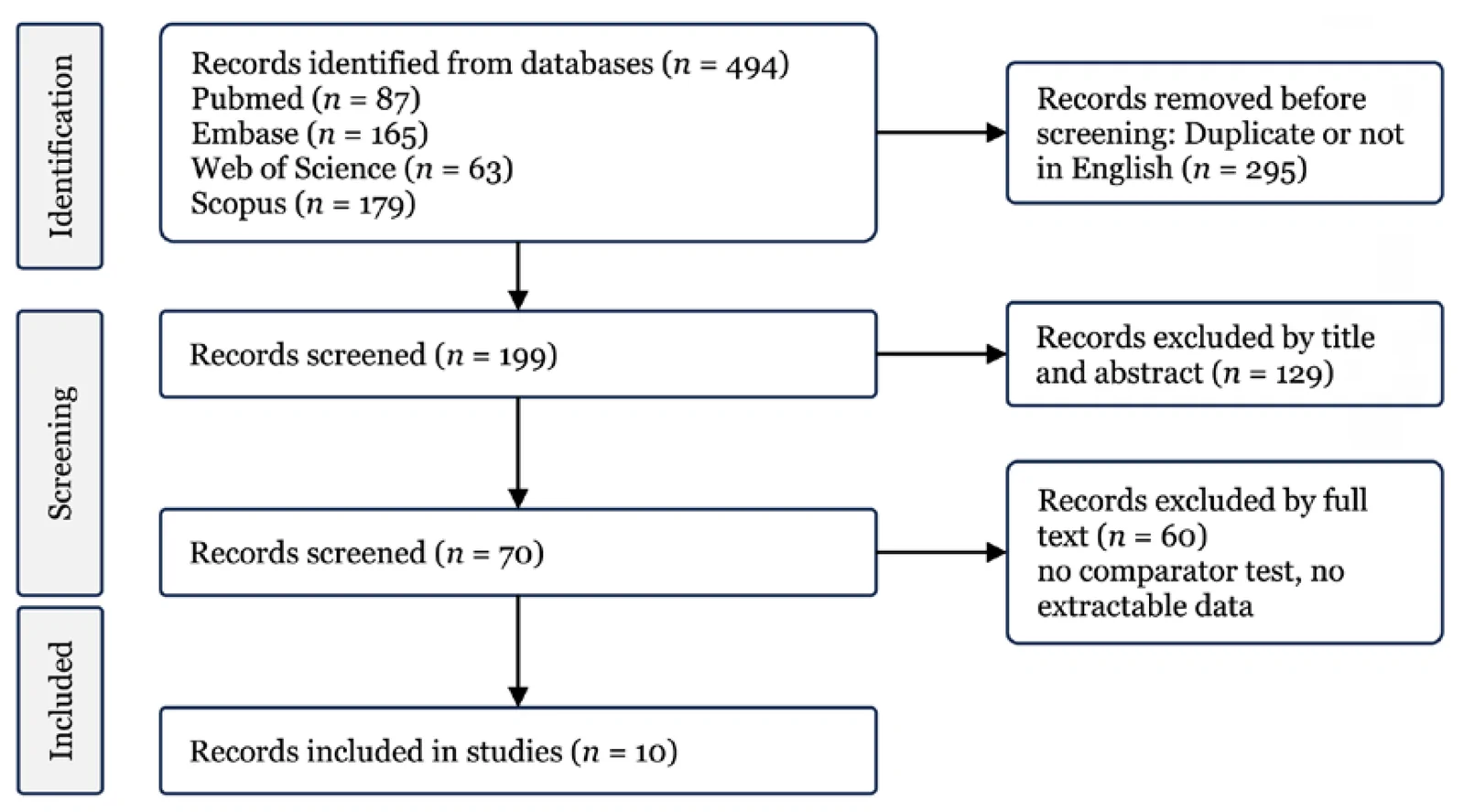
PRISMA flow diagram summarising the literature search, screening, and study selection process

**Figure 2:**
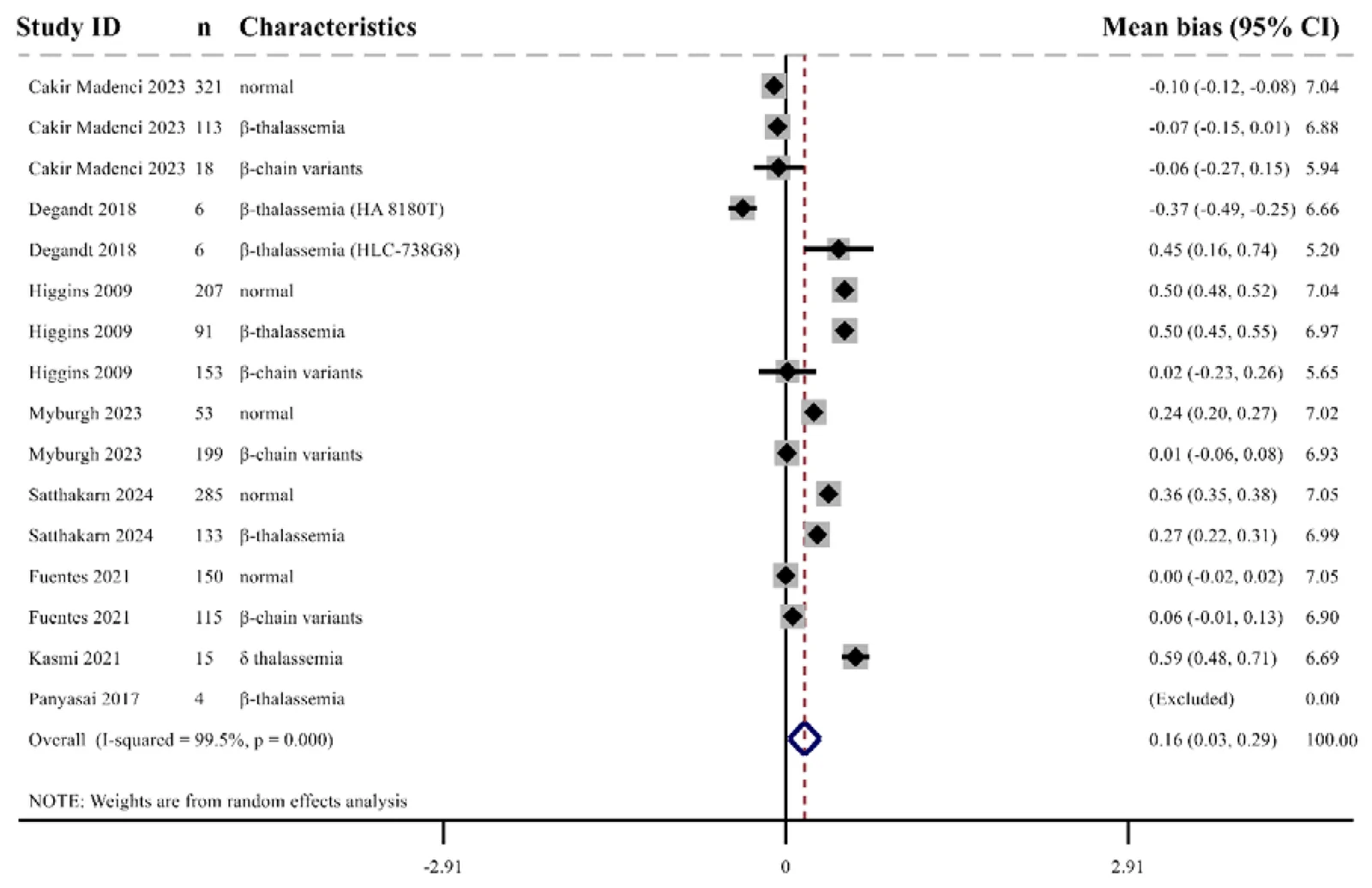
Forest plot of the pooled mean bias between HPLC and CE

**Figure 3:**
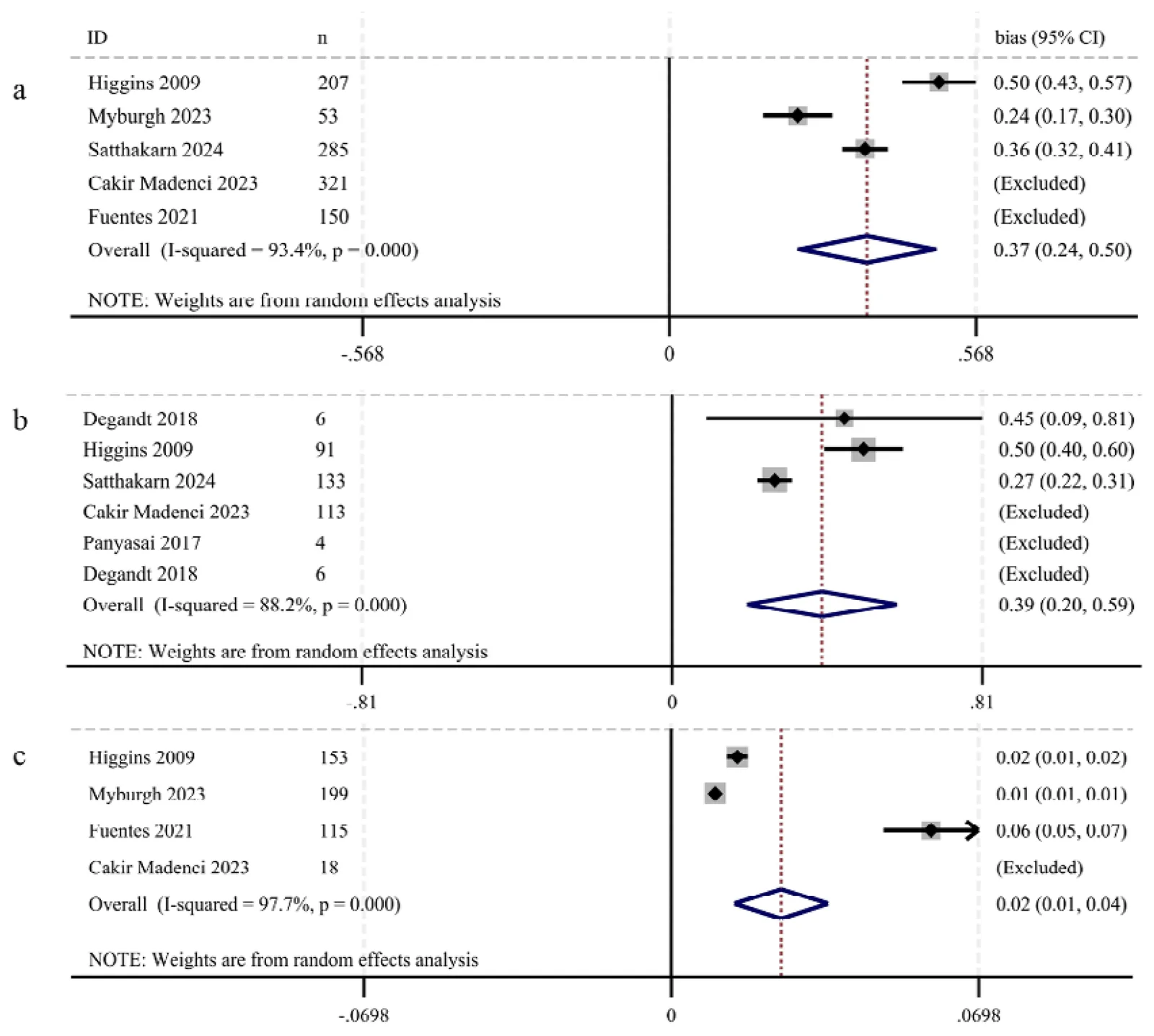
Subgroup analysis of the pooled mean bias between HPLC and CE

**Table 1:** Characteristics of included studies in the meta-analysis comparing HPLC and CE for HbA_2_ quantification

Author (year)	Area	Study design	No.	Population characteristics	HPLC device	CE device
Çakır Madenci (2023) (15)	Turkey	Method comparison	321	Normal	Premier	Capillarys 3
			113	β-thalassemia		
			21	β-chain variants		

Panyasai et al. (2018) (16)	Thailand	Case series	4	β-thalassemia	Variant II	Capillarys 2

Degandt et al. (2018) (17)	Belgium	Cross-sectional	6	β-thalassemia	HA 8180T G8	Capillarys 2

Higgins et al. (2009) (5)	Canada	Cross-sectional	207	Normal	Variant II	Capillarys 2
			91	β-thalassemia		
			153	β-chain variants		

Myburgh et al. (2023) (18)	United Kingdom (UK)	Cross-sectional	53	Normal	Variant II	Capillarys 3
			199	β-chain variants		

Satthakarn et al. (2024) (19)	Thailand	Cross-sectional	285	Normal	Premier	Minicap
			133	β-thalassemia		

Poventud-Fuentes et al. (2021) (20)	US	Cross-sectional	150	Normal	Premier	Capillarys 2
			115	β-chain variants		

Panyasai et al. (2011) (21)	Thailand	Method comparison	6	β-chain variants	Variant	Capillarys 2
Kasmi et al. (2021) (22)	Tunisia	Cross-sectional	15	δ-thalassemia	Variant II	Capillarys 2

Pornprasert and Jaiping (2014) (23)	Thailand	Case series	3	Received blood transfusions from HbE trait donors	Not reported	Not reported

Premier, Premier Resolution (Trinity Biotech, Ireland); HA 8180T, ADAMS A1c HA-8180T (Arkray, Japan); G8, HLC 723G8 (Tosoh Bioscience, Japan); Variant, Variant Haemoglobin (Bio-Rad, US); Variant II, Variant II Haemoglobin (Bio-Rad, US); Capillarys 2, Capillarys 2 Flex Piercing (Sebia, France); Capillarys 3, Capillarys 3 OCTA/TERA (Sebia, France); Minicap, MINICAP Flex Piercing (Sebia, France)
